# Recurrent Network Dynamics; a Link between Form and Motion

**DOI:** 10.3389/fnsys.2017.00012

**Published:** 2017-03-15

**Authors:** Jeroen Joukes, Yunguo Yu, Jonathan D. Victor, Bart Krekelberg

**Affiliations:** ^1^Center for Molecular and Behavioral Neuroscience, Rutgers University, NewarkNJ, USA; ^2^Behavioral and Neural Sciences Graduate Program, Rutgers University, NewarkNJ, USA; ^3^Feil Family Brain and Mind Research Institute, Weill Cornell Medical College, New YorkNY, USA

**Keywords:** early visual processing, recurrent network, motion, form, visual cortex, v1, v2

## Abstract

To discriminate visual features such as corners and contours, the brain must be sensitive to spatial correlations between multiple points in an image. Consistent with this, macaque V2 neurons respond selectively to patterns with well-defined multipoint correlations. Here, we show that a standard feedforward model (a cascade of linear–non-linear filters) does not capture this multipoint selectivity. As an alternative, we developed an artificial neural network model with two hierarchical stages of processing and locally recurrent connectivity. This model faithfully reproduced neurons’ selectivity for multipoint correlations. By probing the model, we gained novel insights into early form processing. First, the diverse selectivity for multipoint correlations and complex response dynamics of the hidden units in the model were surprisingly similar to those observed in V1 and V2. This suggests that both transient and sustained response dynamics may be a vital part of form computations. Second, the model self-organized units with speed and direction selectivity that was correlated with selectivity for multipoint correlations. In other words, the model units that detected multipoint spatial correlations also detected space-time correlations. This leads to the novel hypothesis that higher-order spatial correlations could be computed by the rapid, sequential assessment and comparison of multiple low-order correlations within the receptive field. This computation links spatial and temporal processing and leads to the testable prediction that the analysis of complex form and motion are closely intertwined in early visual cortex.

## Introduction

Form perception is often described as the detection of corners and junctions (Das and Gilbert, 1999), contours (von der Heydt and Peterhans, 1989; Lee and Nguyen, 2001; Ito and Komatsu, 2004), arcs and circles (Hegdé and Van Essen, 2000), and the segregation of figure and ground (Qiu and von der Heydt, 2005). Each of these high-level concepts, however, can also be understood in terms of mathematically precise spatial correlations between three or more points (multipoint correlations). For instance, four-point correlations signal contours (von der Heydt and Peterhans, 1989; Lee and Nguyen, 2001; Ito and Komatsu, 2004), even illusory ones (von der Heydt et al., 1984), and three-point correlations provide information on figure/ground segregation (Victor and Conte, 1991; Yu et al., 2015). This suggests that a framework based on multipoint correlations can be fruitful to understand form perception.

The power of this framework is demonstrated by a cluster of recent findings. First, humans are highly sensitive to the specific multipoint correlations that vary most in natural images (Victor and Conte, 1991; Hermundstad et al., 2014). Because humans are most sensitive for patterns that are least predictable, thus carry most information (Tkacik et al., 2010; Hermundstad et al., 2014), this is evidence for a form of efficient coding (Barlow, 1961; Van Hateren, 1992; Doi and Lewicki, 2014). Second, while only some neurons in area V1 are selective for multipoint correlations, a significant fraction of V2 neurons respond selectively to visually salient three- and four-point correlations (Yu et al., 2015). Moreover, naturalistic textures – which are distinguished from their Gaussian-noise analogs on the basis of multipoint correlations – lead to distinctive responses in V2, both in the human and the macaque (Freeman et al., 2013). In this paper, we propose a novel mechanism by which neurons in V1 and V2 generate such selectivity.

Previous approaches to understand form processing in early visual areas have relied on feedforward models that combine multiple linear filters through static non-linearities (Chichilnisky, 2001; Rust et al., 2004; Simoncelli et al., 2004). In principle, such models can be selective for capturing multipoint correlations. However, as we show below, for the specific dataset we aimed to model, this approach did not fare well. This may in part be due to the poor match between the single-stage feedforward processing in LN models and the multi-stage processing and abundance of recurrent connections in the visual system (see Discussion). We, therefore, developed an alternative approach based on a four-layer artificial neural network with locally recurrent connectivity. This artificial neural network faithfully reproduced neurons’ selectivity for multipoint correlations and generalized beyond the V2 data set that was used to fit the model.

Models – even those with good generalization performance – cannot prove that a certain architectural feature (here recurrent connectivity) is necessary to perform a specific computation (here detection of multipoint correlations). In our view, the true value of a model is that it provides a willing subject that can be probed at length to uncover novel insight into how it computes, and thereby generate novel hypotheses about how the brain may compute (Discussion). Our detailed investigation of the artificial neural network resulted in two major novel insights. First, we found that the model self-organized network elements with response properties similar to individual neurons recorded in V1 and V2, including selectivity for visually salient three- and four-point correlations and surprisingly diverse but characteristic transient and transient-sustained response dynamics. This suggests that these neural dynamics play a role in the detection of complex, static form. Second, the prevalence of transient and transient-sustained response dynamics led us to probe the model, which had been trained only to reproduce the response to static stimuli, with dynamic stimuli. We found that many neurons were tuned for motion and that four-point selectivity was strongly correlated with selectivity for motion. This leads to the testable prediction that complex form analysis and motion tuning are closely intertwined at the single neuron level as early as V1 and V2.

## Materials and Methods

### Experimental Data

The experimental data were obtained using tetrode recordings in areas V1 and V2 of 14 anesthetized and paralyzed macaques. All procedures were approved by the Weill Cornell Medical College Animal Care and Use Committee and were in agreement with the National Institutes of Health guidelines for the humane care and use of laboratory animals.

We recorded 269 neurons in V1 and 153 neurons in V2 and confirmed the recording sites using electrolytic lesions at the conclusion of the experiment. In V1 we classified 32 cells as supragranular, 153 cells as granular and 71 cells as infragranular. In V2 we classified 32 cells as supragranular, 34 cells as granular and 57 cells as infragranular. This dataset consisted of all of the recordings reported in Yu et al. (2015), except for the V1 (13/269) and V2 (30/153) neurons for which laminar identification was uncertain. Details concerning animal preparation, electrophysiological procedures, stimulus alignment, spike-sorting, response analysis, and histology are provided in Yu et al. (2015).

### Visual Stimuli

**FIGURE 1:**
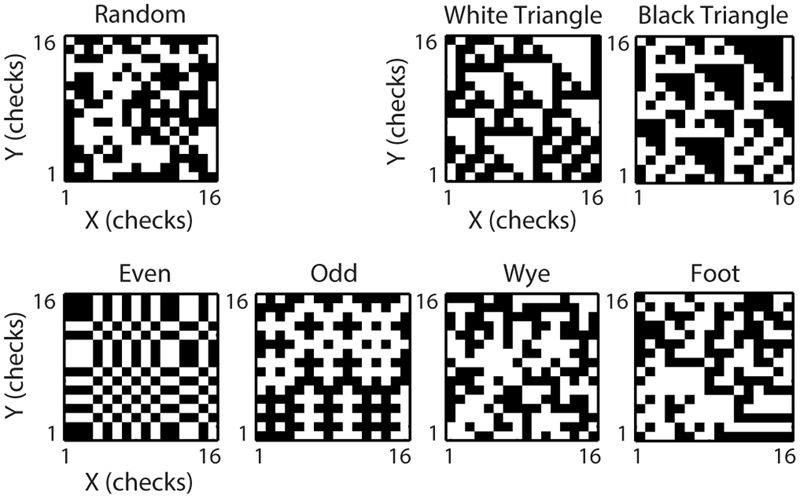
**Multipoint spatial correlation (MSCT) stimuli.** One example texture is shown for each of the texture classes. The visually salient White Triangle Black Triangle textures differ from the random textures in their three-point correlations. The visually salient Even and Odd textures and non-visually salient Wye and Foot textures differ from the Random textures in their four-point correlations.

All stimuli were checkerboards, consisting of a 16 × 16 array of black and white checks. In each experiment, the physical size of the checkerboards was adjusted to match the receptive field of the neuron under study (for details, see Yu et al., 2015). Checkerboards were either random (check colors assigned independently and with equal probability to black or white), or constructed to contain only spatial correlations of a specific spatial configuration and order (**Figure 1**). We call these multipoint spatial correlation textures (MSCT). We studied seven MSCT classes: two classes contained visually salient three-point correlations (*white triangle* and *black triangle*), two classes contained visually salient four-point correlations (*even* and *odd*), two classes contained four-point correlations that are not visually salient (*wye* and *foot*), and one class contained no spatial correlations (*random*). Stimuli were generated via a Markov recurrence rule (Victor and Conte, 1991, 2012). We presented 1024 examples (two repeats each) per MSCT class for 320 ms, interleaved in a pseudorandom sequence. It is important to note that for each MSCT, the specific multipoint correlations are fixed, and there are (on average) no correlations of lower orders (e.g., the *even* stimulus class has a specific fourth-order correlation, but does not have first- (mean luminance), second- (power spectra/spatial frequency content) or third-order correlations). Put differently, these classes form a basis to study the influence of each kind of multipoint correlation.

### Data Analysis

#### Linear–Non-linear Model

In the linear–non-linear (LN) model we adapted from (Chichilnisky, 2001; Rust et al., 2004; Simoncelli et al., 2004) the visual input is first linearly filtered by one or more filters, each filter output is transformed by a static non-linearity, and these outputs are then summed. We used the spike triggered average (STA) and the spike triggered covariance (STC) methods to estimate the filters (Chichilnisky, 2001; Rust et al., 2004; Simoncelli et al., 2004) using the full set of stimuli (1024 examples, 7 classes, 2 repeats) and the mean response over 40–200 ms after stimulus onset. Based on the STA and STC we then estimated the information captured by the maximally informative filters using the iSTAC method (Simoncelli et al., 2004). For display purposes (**Figure 3**), these linear filters were low pass filtered with a 2-dimensional Gaussian (σ = 2 input stimulus checks). Finally, we determined the non-linearity associated with each filter by dividing the histogram of the projected spike triggered ensemble by the histogram of the projected raw stimulus ensemble, over four standard deviations away from the mean. This procedure assumes separability of the filter dimensions (Simoncelli et al., 2004).

We estimated the performance of each LN model separately on the 1024 examples per MSCT class that were used to estimate the LN models (train set) and 10,000 newly generated examples for each MSCT class (test set). For each MSCT stimulus we calculated the model output and averaged the response over all textures in an MSCT class (separately for train and test sets) to obtain an MSCT tuning curve. Model performance was defined as the Spearman correlation between the model tuning curve for the train and test sets, and the experimentally measured tuning curve (based on the train set).

#### Recurrent Form Analysis Model

The two-stage recurrent form analysis model (RFAM) was based on the Elman recurrent neural network (Elman, 1990) implemented in the MATLAB Neural Network Toolbox (version 4). Units in such an artificial network are considered a crude approximation of a neuron or a group of neurons (**Figure 5**). The units were interconnected with adjustable weights simulating synaptic connections with variable strength. Each unit also had an adjustable bias value. The network had one input, two hidden, and one output layer. The input layer consisted of 256 units that each simulated one of the 16 by 16 checks of the experimental stimuli. The input layer was fully connected to the first hidden layer in a feedforward manner. The first hidden layer had 100 units that were fully connected to the 100 units of the second hidden layer, which were fully connected to the output layer of the RFAM, both in a strictly feedforward manner. In addition, the hidden units of both layers were recurrently connected to all hidden units within their layer. The output for each unit (i) was calculated by first determining the weighted sum of its inputs plus the bias value: X_i_ = ∑ _k_w_ik_y_k_ + b_i_, where the index k runs over all units that are connected to unit i, and then passing this through a sigmoid transfer function: y_i_ = 1/(1 + e^-X_i_^).

**FIGURE 2:**
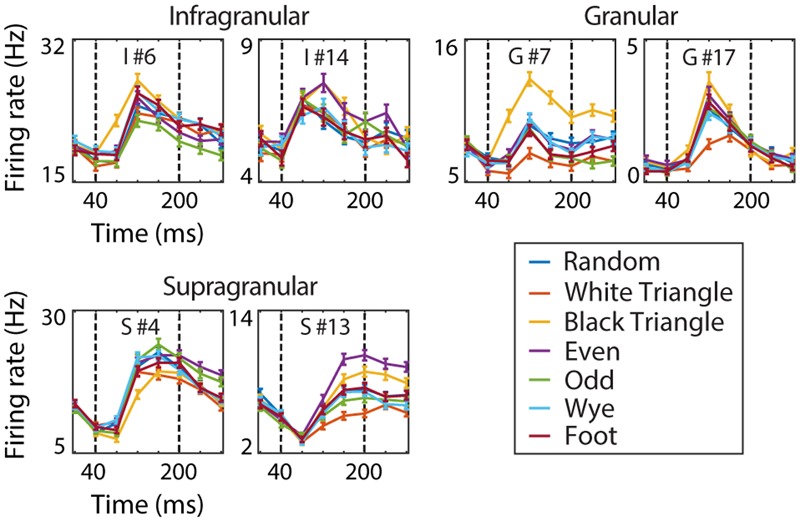
**V2 example cells.** The dynamic response of six V2 example cells to the MSCT textures (mean over 1024 examples and two repeats, error bars reflect the standard error). Dotted vertical lines indicate the time window used for analysis. This figure shows that V2 MSCT selectivity was diverse and that the time course of the neural response contained both transient and sustained components.

We developed two recurrent models. The first (RFAM) was trained to capture the response of all 123 V2 neurons (irrespective of their laminar location) in an output layer with 123 units. In the model network, these output units are not connected; their interaction arises only from sharing a common set of hidden units. **Figure 2** shows six examples of single neuron responses that these output units were trained to capture.

The second RFAM model was trained to capture the average response of the supragranular V2 neurons (the neurons with strongest multipoint tuning). We refer to this model as the RFAM population average; RFAMpa. RFAMpa had a single output unit; after training its activity reproduced the average activity of all V2 supragranular neurons (V2pa).

##### Output patterns

The RFAM was trained to reproduce V2 responses, in the language of artificial neural networks these are called the target patterns, or, because they are the responses of the output units, output patterns. We chose to train the network on what we consider the most interesting phase of the response; the period when selectivity for MSCT arises in most V2 neurons (40–200 ms; see **Figure 2**, marked by the dotted lines). This period excludes the initial descending response of approximately 40 ms that was most likely due to the previous stimulus in the stimulus stream without blank intervals. This period also excludes the response changes that happen on a slower time scale, presumably due to adaptation processes.

Within the period of interest, we binned the spiking response in 40 ms bins to create output pattern sequences of length five. We normalized these responses to a suitable range for the artificial neural network (between zero and one) by first subtracting the minimum firing rate (FR) and then dividing by the maximum FR over all time bins, conditions, and neurons.

##### Input patterns

The input patterns presented to both RFAM designs matched the set of 1024 examples per class used for the electrophysiological experiment. The binary 16 × 16 stimuli were spatially low pass filtered with a two-dimensional Gaussian (σ = 2 checks) to generate a continuous representation, and to approximate the likely input to cortical neurons, which would be filtered by the lens, retina, LGN, and include other sources of blur. Although this low pass filtering introduces second-order spatial correlations in the textures, this is equal for all MSCT classes and does not affect multipoint correlations. Just as in the experiments, the same, static pattern was presented for each of the five 40 ms time bins of a simulated trial. Between trials, the activity in the network was reset to zero to avoid interactions between successively presented training patterns.

##### Training phase

Before training the network, we initialized the weights and bias values of all layers using the method of (Choi et al., 2005). In the training phase, we randomly chose one of the input patterns and presented this to the network and calculated the response of all units in the network for five time steps. Next, we calculated the error as the mismatch between the response of the 123 output units and the 123 V2 cell responses. (For RFAMpa, the error was defined analogously as the mismatch between the single output unit and the V2pa). We then used the backpropagation-through-time algorithm to modify each of the connection weights in the network. This method adjusts each of the weights in the network in negative proportion to its contribution to the error and thereby performs gradient-descent on the high-dimensional error surface. This process was repeated five million times (epochs). Although this optimization method is only guaranteed to find a local and not a global minimum in the error, it works very well in practice, across a wide range of applications (LeCun et al., 2015). In our application, monitoring the error over time showed a steady decline in the error and further training contributed little to a reduction in error (**Figure 6A**). Once the error converged, we froze the network parameters and investigated the trained network.

Because the training algorithm set each of the weights independently, the network had a much larger number of free parameters (>20 k) than traditional models. The potential concern that a model with so many free parameters could “fit anything” was addressed in two ways. First, the parameters were constrained by a large number of output patterns. For the full RFAM network this consisted of >4 million data points (123 neurons^∗^5 time points^∗^1024 input patterns^∗^7 MSCT classes), and even the RFAMpa was trained on >35,000 data points. Second, and most importantly, all measures of performance were based on input patterns that were not used to train the network. In other words, we assessed the network’s ability to generalize its computations to novel, unseen patterns.

As is the case with all artificial neural networks, design choices such as the number of layers, neurons per layer, and number of training epochs proceeded largely by trial and error. For instance, we discarded networks with smaller numbers of hidden units for which the training algorithm failed to converge to a solution. The findings reported here, however, were robust to changes in these choices, and were found reliably in all networks trained on these data (even though each training procedure started from different random initializations of network connectivity).

#### Texture Tuning Index

For a direct comparison of the selectivity of neurons and model output units, we quantified selectivity for MSCT with the mean response over the 1024 experimental examples per MSCT class and time (and two repeats for the V1 and V2 cells). We calculated the texture tuning index (TTI) for each of the six MSCT classes as the absolute value of the Michelson contrast between the mean response to the class (x) and the mean response to the *random* textures: TTIx = |(x – *random*)/(x + *random*)|. A TTI of zero corresponds to no selectivity, higher TTIs represent increasing selectivity.

#### Neural Dynamics

We used principal component analyses (PCA) to investigate the time course of the neural and model unit responses. First, we calculated the mean response over the 1024 experimental examples and the MSCT classes. To align the V1 and V2 responses with the hidden units (which do not have an afferent delay), the former were shifted by their average onset delay (40 ms). All time courses for each neuron and unit were then normalized with a division by the maximum response over time. Next, all hidden units and all V1 and V2 neurons were collected in a single matrix and PCA was applied to this matrix. We quantified the neural dynamics of the hidden units and the neurons by their projection (**Figures 9B,C**) onto the first two principal components (**Figure 9A**).

#### Similarity Analysis

We quantified the similarity between layers in the model and areas in the brain using a linear regression technique previously used to gage similarity between units in a deep neural network model and neurons in inferotemporal cortex (Yamins et al., 2014) and to explore the versatility of neural representations in parietal cortex (Morris et al., 2016).

We first extracted the average response to each of the MSCT classes, for each of the five time bins, for all units in H1 (matrix R^H1^) and H2 (R^H2^). Each of the R matrices had 35 rows (7 MSCT classes ^∗^ 5 time bins) and 100 columns (the number of units). For each neuron in V1 and V2 we also calculated the mean responses to the same input stimuli, in the same time bins, resulting in two matrices with 35 rows and a number of columns that matched the number of neurons in the two samples (R^V 1^,R^V 2^). We then used linear regression to estimate the weights that best captured the neural response as a weighted sum of either the H1or the H2 responses.

Because many of the units in H1 and H2 had similar responses, a standard linear regression that minimizes only the least-squares error, could be ill-constrained. This typically leads to large weights with opposite sign for two units with similar responses. We used ridge-regression to address this. This method simultaneously minimizes the least squares error and a term that penalizes large weights. The ratio between the least squares error term and the penalty term is set by the so-called ridge parameter; it was set to 0.2 here, but all findings were robust across a wide range of ridge parameters.

For each neuron, we then calculated the Pearson correlation between the linear prediction and the actual neural response and we averaged this over all neurons to arrive at a single measure of similarity for the sample of V1 or V2 neurons, and the units in H1 or H2.

For example, to find the best fitting weights describing the V1 population in terms of H2 responses, we first used linear regression to solve for β in the matrix equation:R^V 1^ = R^H2^β. (Each column in the matrix β represents the weights used to approximate a single V1 neuron with a linear combination of H2 units). Then, we determined the correlation between each recorded response [i.e., each column (neuron) of R^V 1^] and its linear approximation in terms of H2 (each column of R^H2^β). We use r^v1,H2^ to refer to the average of these correlations across the sample of neurons in V1. Analogous similarity measures were computed for each of the pairwise combinations of (V1, V2) and (H1, H2).

##### Texture, orientation, speed, and direction tuning

We performed a large set of simulated experiments to probe the response properties of the hidden units. These experiments varied a range of stimulus features: multipoint correlations, orientation, and the direction and speed of translational and rotational motion.

In each experiment, the stimulus was presented for five time steps (200 ms), and the response was averaged over those five time steps and over 10,000 examples from a specific stimulus condition. All selectivity indices were based on these averaged responses (below).

The texture selectivity index (TSIx) was defined as the difference between the average response to the examples from the class (x) and the average response to *random* textures. A positive (negative) TSIe, for instance, means that the unit responded more (less) to the *even* MSCT class than to the *random* class. The magnitude of the index corresponds to the strength of this stimulus preference.

To quantify orientation tuning, we first created oriented stimuli using one-dimensional binary random noise values (16 values) replicated in the *y*-direction (16 values), and then rotated these two-dimensional patterns with one of 18 angles between 0° and 180°. Just as the MSCT patterns these were low pass filtered with a two-dimensional Gaussian (σ = 2 checks). We calculated the orientation selectivity index (OSI) as the largest difference (irrespective of its sign) between the average response to any of the 18 orientations and the average response to all orientations. Hence, a large magnitude of the index represents a strong selectivity for a specific orientation, while the sign of the index indicates whether the unit responds more (OSI > 0) or less (OSI < 0) than average to that orientation.

To quantify motion tuning, we first generated low-pass filtered two-dimensional *random* textures, as described previously. For translational motion, we moved the noise patterns with one of seven speeds (0, 0.5, 1, 2, 4, 8, 16 checks/40 ms) in one of four directions (*upward, rightward, downward* or *leftward*) over five time steps (200 ms). For the typical RF size of around 1° and a time step of 40 ms these simulated speeds correspond to 0–25°/s. For rotational motion, we rotated the noise patterns with one of nine speeds (0, 0.5, 1, 2, 4, 8, 16, 32, 64, 128°/40 ms) in one of two directions (*clockwise* or *anti-clockwise*) over the five time steps.

The speed selectivity index was calculated separately for translational and rotational motion (SSIt, SSIr), and defined as the largest difference (irrespective of its sign) between the average responses to any of the seven speeds and the average response to the stationary condition. Hence, a large magnitude of the index represents a unit that responds very differently to moving stimuli (at some speed) than to stationary stimuli.

To calculate the direction selectivity index, we first determined the speeds that evoked the largest response across all directions (preferred speed) and the speeds that evoked the smallest response across all directions (anti-preferred speed). The DSI was then defined as the largest difference between the average response to any of the four directions of motion at either the preferred or anti-preferred speed. This was calculated separately for translations (DSIt) and rotations (DSIr).

## Results

We combined experimental and computational methods to gain insight into early visual computations underlying complex form processing. After giving an overview of the experimental data, we first present an attempt to capture the computational principles underlying tuning for multipoint correlations using an established method based on feedforward processing. This method has previously been used to describe the computations in a wide range of visual processing stages, including, for instance, the retina (Chichilnisky, 2001), V1 (Rust et al., 2005), and the middle temporal area (Richert et al., 2013). As we will show below, however, that approach fails to explain the data at hand. This motivated us to develop a novel approach using a recurrent network model, which is the focus of the third and major part of the Results.

### Experimental Data

We recorded from 269 neurons in anesthetized, paralyzed macaque V1 and 153 neurons in V2. Based on histological verification we classified 32 V1 cells as supragranular, 153 cells as granular, and 71 cells as infragranular. In V2 we classified 32 cells as supragranular, 34 cells as granular, and 57 cells as infragranular (Yu et al., 2015). This dataset consisted of all recordings reported in Yu et al. (2015), except for the V1 (13/269) and V2 (30/153) neurons for which laminar identification was uncertain. We recorded their responses to 1024 example textures of seven texture classes that isolate multipoint correlations previously studied psychophysically (Victor and Conte, 1991, 2012; Tkacik et al., 2010; Hermundstad et al., 2014). Examples of the MSCT are shown in **Figure 1**. For the *random* textures, check colors were assigned white or black independently. The *white triangle* and *black triangle* textures contain perceptually salient three-point correlations and the *even* and *odd* textures contain perceptually salient four-point correlations (Hermundstad et al., 2014). Finally, the *wye* and the *foot* textures contain four-point correlations that are not perceptually salient (Victor and Conte, 1991). All textures were scaled and then presented inside the classical receptive field of the neuron under study (Yu et al., 2015).

Yu et al. (2015) showed that the MSCT evoke robust, but complex dynamic responses (**Figure 2**), and that some V1 and many V2 cells showed selectivity for MSCT. To obtain a robust measure of this selectivity, we determined an MSCT tuning curve (the average response to the example stimuli from an MSCT class), separately for two randomly chosen halves of the data (512 examples per class each). For each randomly chosen 50/50 split we calculated the correlation between the two tuning curves and repeated this process 5000 times (drawing new random 50/50 subsets each time). We defined consistency as the mean of the distribution of correlations over these 5000 sets. A neuron with multipoint tuning that generalized to all examples of the MSCT classes would have a consistency of one. In V1, the consistency quartile range [25th percentile, 75th percentile] was [0.06, 0.42], in all of V2 it was [0.33, 0.60], and in supragranular V2 it was [0.46, 0.70]. This shows that a substantial fraction of neurons, and especially those in the supragranular layers of V2, have robust tuning for multipoint correlations. For other measures of tuning and a detailed analysis of the multipoint tuning in V2 neurons, we refer to (Yu et al., 2015). Our goal here was to uncover computational principles that could underlie the tuning for multipoint correlations observed primarily in V2.

### Linear–Non-linear Model

In an LN model each subunit receives the same input, which is passed through a (linear) filter and then through a static non-linearity. Here we used the information theoretic spike triggered average and covariance analysis (iSTAC) method (Pillow and Simoncelli, 2006) to estimate the most informative subunit filters as well as their non-linearities (see Materials and Methods). The input to the iSTAC method was the collection of 1024 example textures for each of the seven MSCT classes and the output was the mean FR evoked by the neuron in a time window 40–200 ms after stimulus onset. We note that the iSTAC procedure does not attempt to determine the dynamics of the initial linear filters and that there is a second linear stage that simply sums across each LN component to generate a single output based on multiple, parallel LN pathways. Because this second stage has no additional free parameters, we refer to the model as an LN model (even though it could technically be considered an LNL model).

**FIGURE 3:**
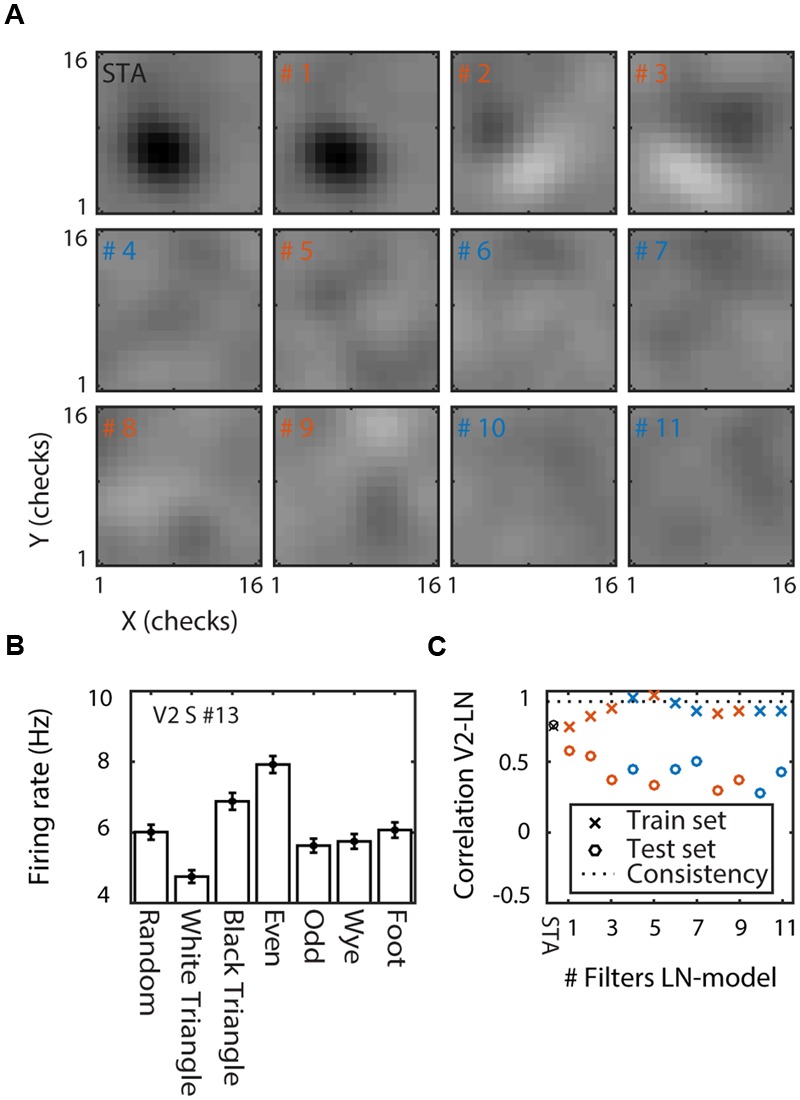
**Linear–non-linear model of a V2 supragranular example cell (#13). (A)** Linear filters. The spike triggered average (STA) and the 11 linear filters ordered by the amount of information they carry (filter numbers show rank order). Red/blue indicates filters that increase/decrease firing rate above/below the mean of the cell. **(B)** MSCT selectivity. Mean response to the seven MSCT classes. Error bars indicate standard error over examples. This supragranular V2 cell responded selectively to three- and four-point textures. **(C)** Performance of the LN model. Correlation between the neuron’s texture tuning curve and the tuning curve of the LN models with increasing number of filters (*x*-axis). Performance is shown separately for the train set (crosses) and for generalization to a test set (open circles). The dotted line shows the tuning consistency of the V2 neuron (see main text for details). This figure shows that the LN model captured the tuning in the train set but not the ability of the V2 neuron to generalize across examples of the MSCT classes.

**Figure 3A** shows the STA and the eleven most informative filters in the space spanned by the STA and STC (Pillow and Simoncelli, 2006) for one of the V2 supragranular cells (#13; **Figure 2**). The STA shows that this cell has a polarity-sensitive patch in the center of the cell’s RF. The similarity between the STA and filter #1 (**Figure 3A**) shows that most of the information was carried by the STA. The next two most informative filters (#2 and #3, **Figure 3A**) had orthogonal orientation sensitivity. In contrast to filter #1, the orientation sensitivity was polarity insensitive; stimuli that matched the filter or its polarity inverse evoked increased responses. The next eight filters were excitatory (#5, #8, and #9; textures that match these filters increased the FR) or suppressive (#4, #6, #7, #10, and #11; textures that match these filters decreased the FR), but none had obvious spatial structure.

In the feedforward view of visual processing, this set of filters and their corresponding non-linearities, accounts for the output of a V2 cell. We asked whether this model could explain sensitivity for complex form. **Figure 3B** shows the neuron’s MSCT tuning curve (mean response over 1024 examples, two repeats, and time window 40–200 ms after stimulus onset). The neuron was selective for MSCT (ANOVA, *p* < 0.0001). Specifically, compared to the *random* textures, its response was smaller for three-point *white triangle* textures (*post hoc t*-test, *p* < 0.0001), but larger for *black triangle* textures (*post hoc t*-test *p* < 0.008), and four-point *even* textures (*post hoc t*-test *p* < 0.0001). The consistency of MSCT tuning (see above) for this neuron was very high: *r* = 0.93, showing that its tuning generalized almost perfectly to all examples of the MSCT classes.

We quantified the LN model’s ability to reproduce MSCT tuning as the Pearson correlation between its MSCT tuning curve and the MSCT tuning curve of the corresponding neuron. This performance was first calculated based on the response to the 1024 examples per MSCT class that were also used to estimate the LN model (train set; training tuning curve; training performance). To assess the model’s ability to generate consistent MSCT tuning for stimulus examples that were not part of the training set, we also generated a tuning curve based on the simulated response to 10,000 new examples per MSCT class (test set; generalization tuning curve, generalization performance). The correlation between the generalization tuning curve and the neural tuning curve defined the generalization performance. To assess the contribution of each of the filters, we calculated model performance separately for models that included only the STA, only the first (most-informative) filter, only the first two most informative filters, up to the first fifteen most informative filters.

**Figure 3C** shows the performance of the LN model for the train set (crosses; training performance) and the test set (open circles; generalization performance). For this neuron, the STA model captured a considerable amount of the MSCT selectivity (*r* = 0.76), and a five-filter LN model resulted in almost perfect training performance (*r* = 0.98). However, the model fared poorly on new example textures, with generalization performance around *r* = 0.5 regardless the number of filters in the model.

For comparison, the dashed line in **Figure 3C** shows the generalization performance of the example neuron (*r* = 0.93); clearly the LN model performed much worse than the example neuron, suggesting that many of the filters and corresponding non-linearities did not capture the underlying regularity of texture tuning.

**FIGURE 4:**
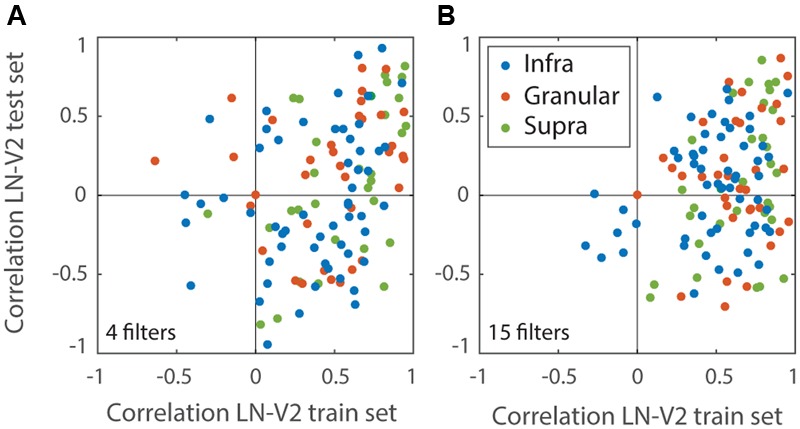
**Performance of the LN models.** Performance measures the correlation between MSCT tuning of each V2 neuron and its LN Model. Performance was calculated separately for a set of novel textures (test set) and plotted against the performance on the stimulus set that was used to estimate the LN model (train set). Each dot represents a single V2 neuron. Colors represent laminar origin of the neurons (see legend). **(A)** LN models based on the first four most-informative filters. The selection of four filters was based on the population average generalization performance on the test set; this was best for four filters, suggesting that additional filters mainly captured noise. **(B)** LN models based on the first fifteen most-informative filters; these models had good performance on the train set but generalized poorly. This figure shows that the LN models captured a significant fraction of the variance on the training set, but even when restricted to the best set of filters **(A)** generally failed to generalize to novel textures.

We estimated analogous LN models for each of the V1 and V2 cells. These models often explained a sizeable fraction of the measured V1 and V2 MSCT selectivity in the training set (V1 mean *r* = 0.56 ± 0.3 SD; V2 mean = 0.57 ± 0.28 SD), but they did not generalize to new stimulus patterns drawn from the MSCT classes (even for cells that had highly consistent MSCT tuning). **Figure 4** documents this for the V2 population, separately for models that included only the first four filters (which on-average had the best generalization performance) and models that included 15 filters.

The lack of out-of-sample generalization implies that these LN models provided little insight into the computations underlying sensitivity to multipoint correlations and demonstrates the need for a different approach. While it is possible that a more complex feedforward network could be designed to capture form selectivity, we instead chose to pursue a model with locally recurrent connections. Our primary reason for this is that such networks have the potential to capture neural dynamics in a natural manner, and because recurrent connections are an ubiquitous feature of cortex with a poorly understood function (see Discussion).

### Recurrent Form Analysis Model

Previous work has shown that recurrent connections can endow a network with a powerful ability to compute complex functions of its inputs (Quiroga et al., 2016), and more specifically, capture higher-order space-time correlations that underlie motion perception (Joukes et al., 2014; Pachitariu and Sahani, 2017). This, together with the fact that recurrent connections are ubiquitous in cortex led us to the hypothesis that a recurrent network could also be a basis for complex form analysis.

**FIGURE 5:**
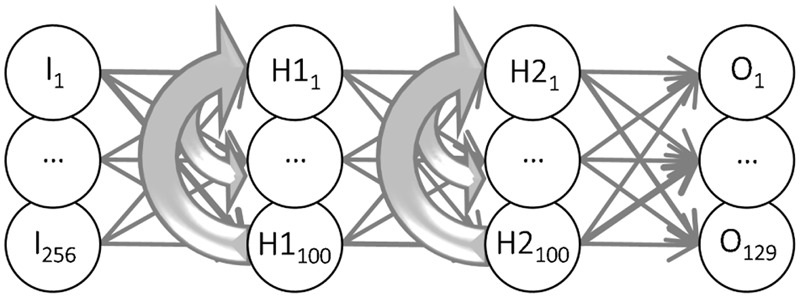
**Recurrent Form Analysis Model (RFAM).** The two -stage recurrent neural network had 256 input units (one for each check in the stimuli shown in **Figure 1**) that were all-to-all connected to the units of the first hidden layer. They, in turn, were all-to-all connected to the units of the second hidden layer. Both hidden layers had 100 units, which were recurrently connected to all units within the same layer (thick gray lines). The units of the second hidden layer were all-to-all connected to the output unit(s). The weights of the connections between the neurons were adjusted in an iterative procedure (backpropagation through time) until the 123 output units reproduced the dynamic response of each of the 123 V2 neurons.

We investigated this hypothesis with a recurrent network consisting of (artificial) neurons, all with identical intrinsic properties and connections with modifiable weights (Elman, 1990). The recurrent neural network (**Figure 5**) had 256 input units; one per check in the MSCT textures. The input units were connected in a feedforward manner to a first hidden layer (H1, 100 units) and the units in H1 were feedforward connected to the second hidden layer (H2, 100 units). H2 units in turn connected feedforward to each of the 123 output units. Recurrent connections were introduced within H1 (each H1 neuron connected to all other H1 neurons) and, analogously, within H2.

In a single step (epoch) of the network training procedure, we first presented one of the 1024 experimental example textures per MSCT class and simulated the response of the output units. Second, we calculated the mismatch between the recorded neural response of the 123 V2 neurons and the observed simulated response. This mismatch was used as the error signal in the back-propagation-through-time algorithm to adjust the weights of all connections in the network (see Materials and Methods). Connection weights were constrained only by the training algorithm and could take on positive (excitatory) or negative (inhibitory) values. We refer to this model as the RFAM. After training the network, the first step was to investigate whether RFAM could reproduce both the MSCT selectivity and the temporal dynamics of the recorded neural responses.

**FIGURE 6:**
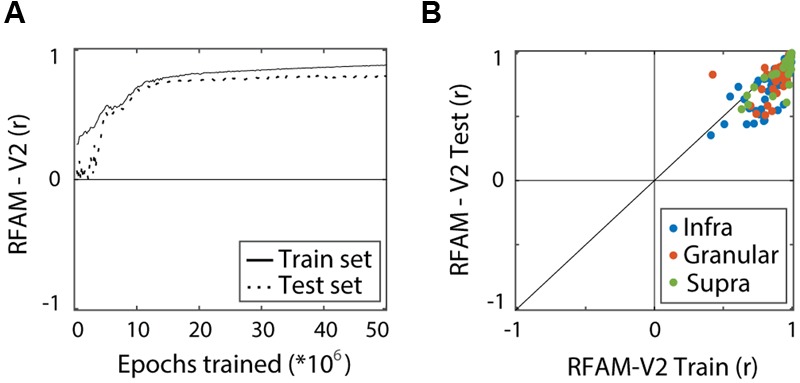
**Recurrent Form Analysis Model training and performance.** Performance measures the correlation between MSCT tuning of the RFAM output units and the tuning of the target V2 neurons. Performance was calculated separately for the experimental stimulus set that was used to train RFAM (train set) and a set of novel textures (test set). **(A)** Average performance of the 123 RFAM output units. High performance was reached for both the train set (solid line) and the test set (dashed line). **(B)** Comparison of the performance of RFAM on the train set and the test set. Each dot represents a single V2 neuron. Colors represent laminar origin of the neurons (see legend). This figure shows that RFAM captured texture tuning in V2 neurons, and generalized to new examples from the texture classes.

We quantified performance of the RFAM output units separately for the train set (1024 experimental examples per MSCT class) and for a generalization set (10,000 new examples per MSCT class), just as we did for the LN model. **Figure 6A** shows how the performance (averaged over all 123 output units) improved with training. After five million training epochs, RFAM captured the MSCT tuning for textures in the train set (solid lines, *r* = 0.88) as well as textures that were not used to fit the model (dotted lines, *r* = 0.81). **Figure 6B** shows the performance of each of the RFAM output units on the train set plotted against the performance on novel textures that were not used to train the model (test set).

Taken together these results show that the RFAM model captured the essence of MSCT tuning observed in individual V2 neurons. Most importantly, and in contrast to the LN model, generalization to the out of-sample test set was only slightly worse than the performance on the training set. This suggests that two layers of recurrently connected neurons are sufficient to generate the tuning for multipoint correlations observed in V2.

### Population Average RFAM

**FIGURE 7:**
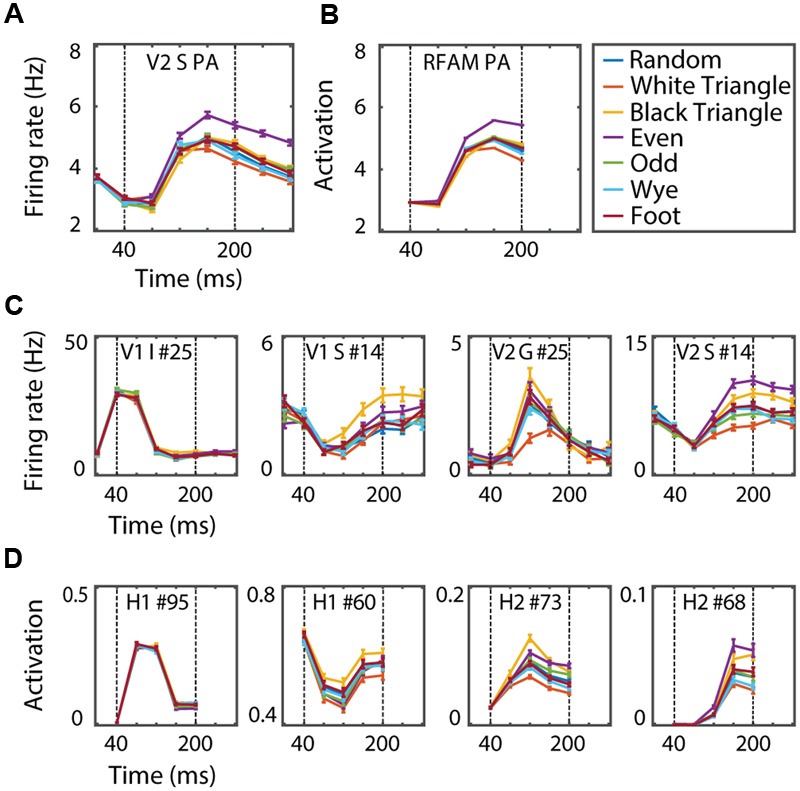
**(A)** Dynamics of the V2pa response, averaged over the 1024 examples per MSCT class. **(B)** Dynamics of the RFAMpa response, averaged over the same examples. **(C)** Dynamics of two V1 and two V2 neurons. **(D)** Dynamics of two H1 and two H2 RFAMpa units. Error bars indicate standard error over examples. This figure shows that, even though RFAMpa was trained only on static MSCT and only to reproduce the V2 population average responses, the hidden units self-organized diverse and complex MSCT selectivity and time courses that were also observed in V1 and V2 neurons.

In the analysis so far, we used the responses of each V2 neuron to train the RFAM and the LN models, including cells that had weak MSCT selectivity or low consistency over examples of an MSCT class. This allowed for a direct assessment of the models’ ability to capture all experimental data. However, our main interest is not the specific observed texture tuning based on a subset of examples for any given MSCT class, but rather the underlying tuning rule for the full MSCT class. Combined with the goal to stay close to the experimental data, we chose to approximate this ideal with the average response of the 32 V2 supragranular cells (**Figure 7A**). We refer to this population average as V2pa. The V2pa had a high consistency (*r* = 0.89; see Materials and Methods) indicating robust and consistent selectivity for all examples drawn from the MSCT classes. We modeled the V2pa with a single RFAM output unit (RFAMpa). For each of the 1024 example textures used in the experiment the target output used in the learning rule was the mean response of the V2pa across all 1024 textures of the same class used in the experiment. Put differently, V2pa and its model RFAMpa embody the consistent MSCT selective response observed on average in the supragranular layer of V2.

After training, RFAMpa had a strong preference for the *even* texture class and it responded with a transient-sustained response, just as the V2pa (**Figure 7A**). Most importantly, texture tuning of the RFAMpa network generalized well to textures not used in the training process (**Figure 7B**, train set *r* = 0.98, generalization *r* = 0.88). This shows that the RFAMpa solves the same computational problem that the supragranular V2 population solves; it consistently detects multipoint correlations in static images. Our next goal is to investigate how the model computes, and use this to generate a hypothesis and experimentally testable predictions for the analogous computations in the brain.

To answer the question how the recurrent network computes we analyzed the response properties of the hidden units. By focusing on a network that has been trained to produce a single output, we know that (by construction) the only goal of each hidden unit’s response is to bring the output unit closer to its target. This greatly simplifies the interpretation of hidden unit response properties and is a major advantage over analyzing the hidden units of the full RFAM network with 123 output units, in which each output unit has a slightly different target, and all hidden units contribute to each of those computations to some extent. Nevertheless, at the end of the Results section we will return to the full RFAM network and show that the salient properties of its hidden units match those of the RFAMpa model.

#### Hidden Units: Texture Tuning Properties

**FIGURE 8:**
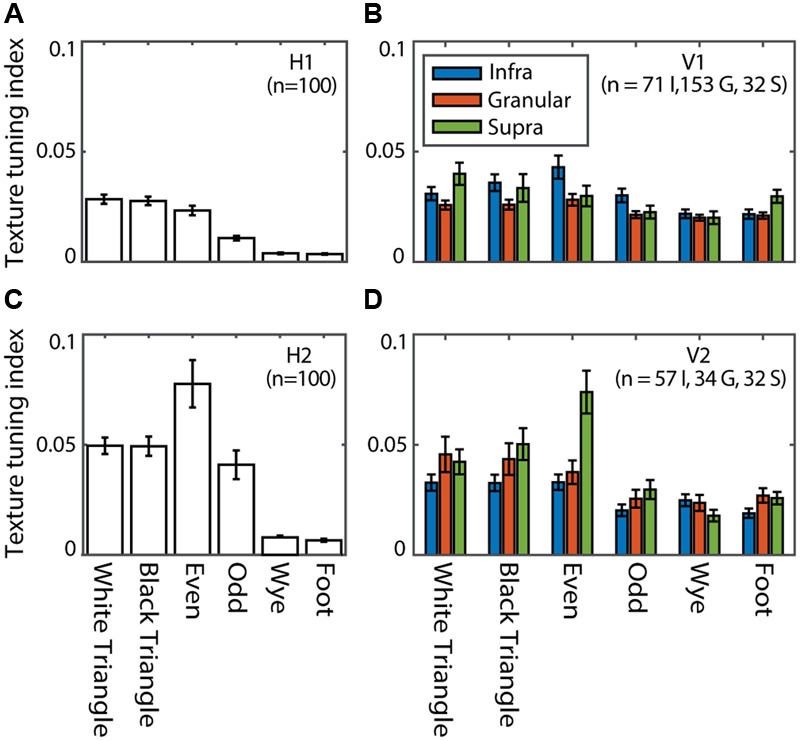
**Texture tuning in the RFAMpa hidden layers (H1, H2) and V1, V2.** Texture tuning quantified the average difference in response to examples from a given MSCT class and the random class. **(A)** Mean texture tuning for the six MSCT classes for the units in H1 of the RFAMpa. **(B)** Mean texture tuning of V1 neurons, grouped by cortical layer (legend). **(C)** Same as **(A)**, now for H2 **(D)** Same as **(B)**, now for V2. Error bars indicate standard error over units/neurons. Texture tuning was particularly strong for the visually salient MSCT (white triangle, black triangle, even, and odd) and stronger for H2 than H1. This qualitatively matches the MSCT selectivity properties of V1 and V2 neurons.

We define a TTI as the relative change in average response to one of the MSCT classes compared to the *random* class (see Materials and Methods). **Figure 8** shows the TTI for each MSCT class, averaged over V1 neurons (**Figure 8B**) and V2 neurons (**Figure 8D**). This analysis confirms (using a slightly different metric) the results of (Yu et al., 2015); textures with visually salient high-order structure lead to responses distinct from those evoked by *random* textures, particularly in the supragranular layers of area V2. **Figures 8A,C** show the equivalent TTI for the hidden units of the RFAMpa. In the first hidden layer (H1), TTI’s were modest (**Figure 8A**), but the second hidden layer (H2, **Figure 8C**) had substantial texture tuning, in particular for those textures that are perceptually salient (*white triangle, black triangle, even*, and *odd*). There was little selectivity for the perceptually non-salient four-point textures (*wye, foot*) in either H1 or H2.

Taken together, this analysis shows that, although the only task given to the RFAMpa output unit was to reproduce the response of the V2pa (**Figure 7A**) at the population level, the training algorithm produced a network with hidden units whose MSCT selectivity was similar to that observed in V1 and V2 neurons. Note that the full range of V2 tuning properties is produced by the H2 units, even though the V2pa response was primarily selective for the *even* texture class, and showed little if any tuning for the other classes (see Discussion).

Thus far, we only analyzed the time-averaged responses. The V1 and V2 cells, however, had characteristic transient and/or sustained response properties (**Figure 2**). One of the main advantages of a recurrent network is that it can capture such dynamics more naturally than a feedforward model, and indeed, the RFAM and RFAMpa models were trained to reproduce the full time course of the response, not just the mean. This allowed us to investigate whether these dynamics play a role in generating selectivity for static stimuli with multipoint correlations.

#### Hidden Units: Temporal Dynamics

**Figure 7D** shows the time course and MSCT selectivity of sample units in H1 and H2 and example neurons in V1 and V2 with similar tuning and response dynamics. Across the population of H1 and H2 units we observed a large variety of texture preferences and response dynamics. Notably, these preferences or dynamics could be quite different from those of the output unit (and V2pa). For instance, H1 unit #95 had almost no MSCT selectivity but a transient time course. H1 unit #60 and H2 unit #73 responded most strongly to *black triangle* textures; neither of these properties match the V2pa or RFAMpa. Similar properties, however, were observed in the individual V1 and V2 neurons. For instance, **Figure 7C** shows two V1 neurons (first two panels) and two V2 neurons (last two panels) with response properties that qualitatively match the H1 and H2 units in the row below. These examples were hand-picked, but the following formal analyses confirmed a high degree of similarity between the dynamics of H1 and H2 on the one hand and V1 and V2 on the other.

**FIGURE 9:**
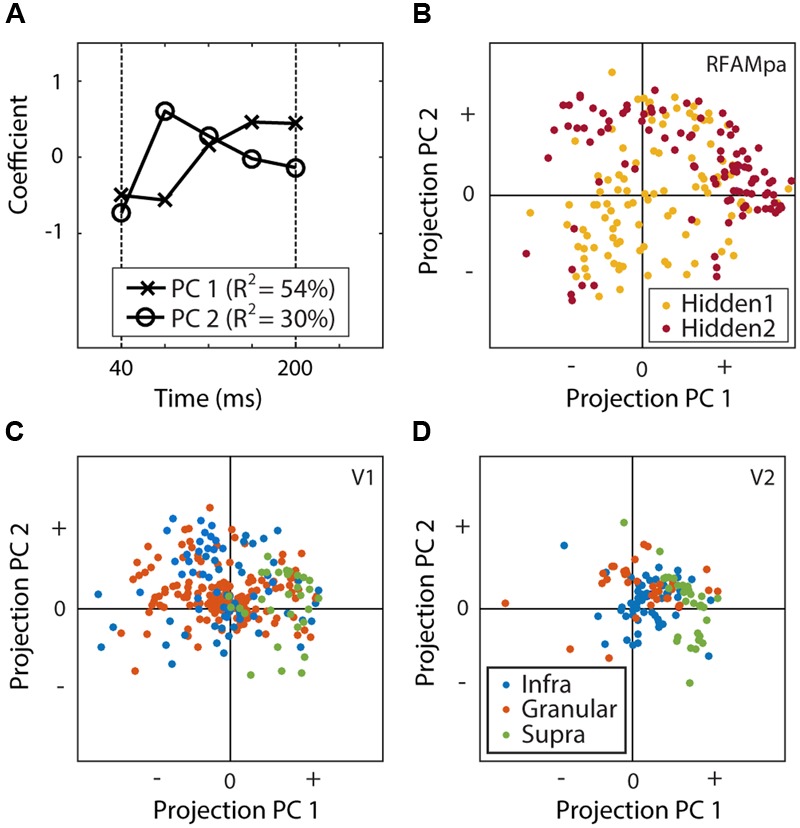
**Principal component analysis of the time courses. (A)** The first two principal components (PC1 and PC2) explained 84% of the temporal response properties for all units and cells. **(B–D)** Projections of the responses of the units of H1 and H2 **(B)**, V1 **(C)**, and V2 **(D)**. This figure shows that the response dynamics as well as the hierarchical organization in V1 and V2 are at least qualitatively similar to those found in the recurrent network model.

We used principal component analysis (PCA) on the dynamics of all units (H1, H2) and all neurons (V1, V2) to extract a common basis for a low-dimensional description of the dynamics (see Materials and Methods). Two components explained 84% of the variance in the temporal dynamics (**Figure 9A**), showing that little information is lost when describing each neuron by two numbers (the projections onto these two components). **Figures 9B–D** displays each of the subpopulations in this coordinate system and allows for a visual comparison and qualitative interpretation. First, H1 and H2 clusters overlap with the V1 and V2 clusters, showing that their dynamics were generally similar. More quantitatively, 84% of the convex hull of V1 (**Figure 9C**) and V2 (**Figure 9D**) overlapped with the convex hull of H1 and H2 (**Figure 9B**).

The figure also suggests that a modest degree of hierarchical organization is reflected in the neural response dynamics; the V2 neurons had more positive projections onto PC1 than the V1 neurons. We quantified this using a two-way ANOVA with layer and area as factors and the projection on PC1 as the dependent variable. The main factors of area *F*(1,373) = 8.93; *p* < 0.01) and layer [*F*(2,373) = 45.46; *p* < 0.001] were both significant, but their interaction [*F*(2,373) = 2.06; *p* > 0.1) was not. Consistent with this (specifically, the main effect of area), the projections of H2 response dynamics onto PC1 were also more positive than those of H1 (**Figure 9B**; *t*-test *p* < 0.001).

We also quantified the match between model units in H1 and H2 and neurons in V1 and V2 with a similarity analysis based on linear regression (Yamins et al., 2014; Morris et al., 2016). The idea behind this analysis is that if the responses in the H1 set are more similar to those in the V1 set than the V2 set, then fitting V1 responses using the weighted linear sum of H1 responses should produce a better match with actual V1 responses than fitting V1 responses using the weighted linear sum of H2 responses. We used linear (ridge) regression to find the best fits and defined similarity as the correlation between the fitted and actual neural responses (see Materials and Methods). This analysis showed that H1 was most similar to V1 (r^V 1,H1^ = 0.52) but H1 could not capture the response dynamics of V2 as a whole (r^V 2,H1^ = 0.08) or the supragranular neurons in V2s (r^V 2s,H1^ = 0.02). The H2 units were most similar to the V2s population (r^V 2s,H2^ = 0.63), less similar to the V2 population as a whole (r^V 2,H2^ = 0.38) and least similar to the V1 population (r^V 1,H2^ = 0.29). In other words, even though neither H1 nor H2 was a perfect description of V1 or V2 (correlations in the 0.5–0.6 range), the hierarchical organization of form information processing was reflected in the fact that H1 captured V1 best and H2 captured V2s best.

This analysis shows a surprising level of generalization: the RFAMpa network was tasked only with reproducing the average time course of the V2pa (**Figures 7A,B**) in response to the MSCT patterns and never trained on the individual responses of V1 or V2 neurons. Nevertheless, the V1 and V2 time courses were captured reasonably well by a linear combination of the H1 and H2 units, respectively. We take this as evidence to support our claim that the complex dynamic responses, a wide range of MSCT tuning profiles, and their hierarchical organization are essential components of complex form analysis in early visual cortex (see Discussion).

#### Hidden Units: Motion and Form Tuning Properties

The rapid and transient dynamics of the hidden units suggest that they could play a role in the detection of moving patterns. To investigate this, we performed simulated experiments to measure tuning curves for multipoint texture class, orientation, the speed of translational or rotational motion, and the direction of translational or rotational motion (see Materials and Methods).

**FIGURE 10:**
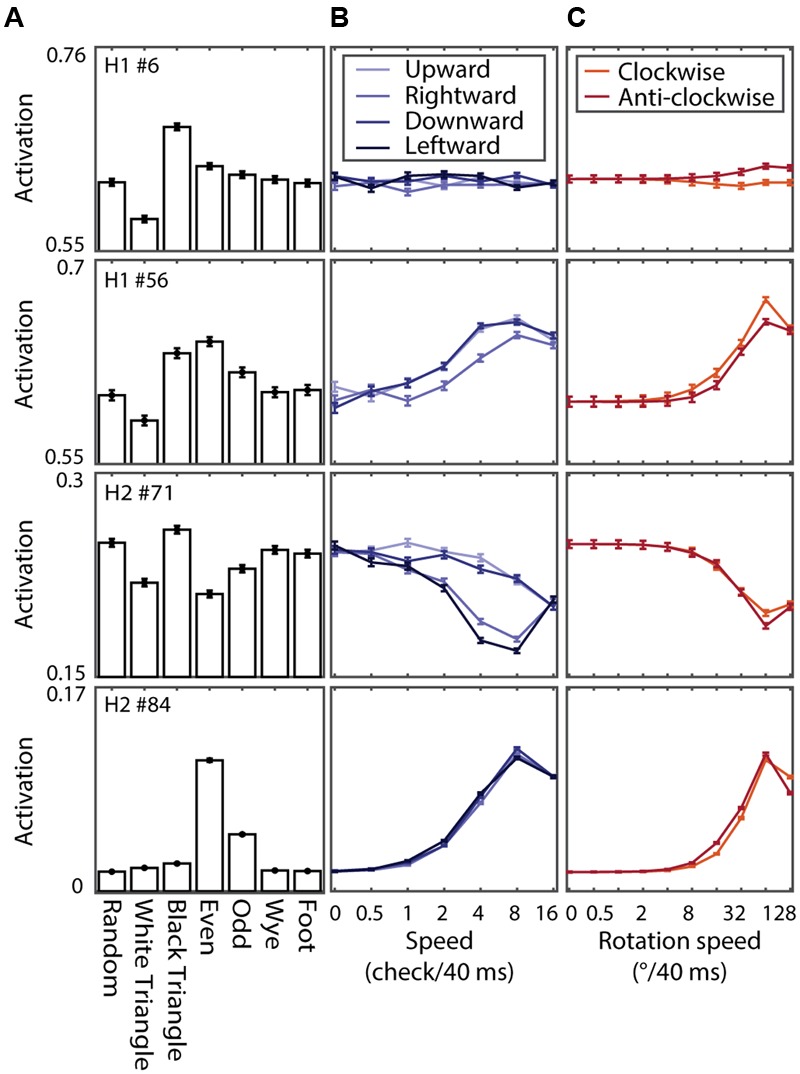
**Texture and motion tuning of four example units in RFAMpa. (A)** Texture tuning. Mean responses to examples from the seven MSCT classes. **(B)** Motion tuning. Response to translating binary random noise patterns at different speeds (*x*-axis) and directions (colors indicate direction of motion as show in the legend). **(C)** Same as **(B)**, now for rotational motion. Error bars indicate standard error over examples. This figure shows that, although RFAMpa was trained only on static MSCT and only to reproduce the V2 population average responses in **Figure 7A**, it self-organized hidden units with diverse selectivity for the speed and direction of moving texture patterns.

**Figure 10** shows the tuning curves of four example units. The different panels show the tuning for MSCT classes (**Figure 10A**), translational speed and direction (**Figure 10B**), and rotational speed and direction (**Figure 10C**). These example units show the range of tuning across the two hidden layers, from virtually no effect of motion (example unit in the first row), band-pass speed tuning with modest direction tuning (second row), low-pass speed tuning with substantial direction tuning for translations but not rotations (third row), and high-pass speed tuning (fourth row). Note that this tuning, of a magnitude similar to the tuning for textures (**Figure 10A**), emerged even though the network was never exposed to any moving pattern during the training phase.

An interesting clue about the computations performed by the network comes from comparing the MSCT selectivity (the true goal of the network) to the motion and orientation tuning strength (emergent properties of the network). We defined indices to quantify selectivity in each of these feature dimensions. The texture selectivity index, TSI, for instance, represents a hidden unit’s preference for textures from one of the MSCT classes compared to the random class. The speed selectivity indices for translation (SSIt) and rotation (SSIr) represent a unit’s preference for moving compared to stationary stimuli, and the direction selectivity indices for translation (DSIt) and rotation (DSIr) represent a unit’s preference for one direction compared to the other directions (see Materials and Methods for details).

**FIGURE 11:**
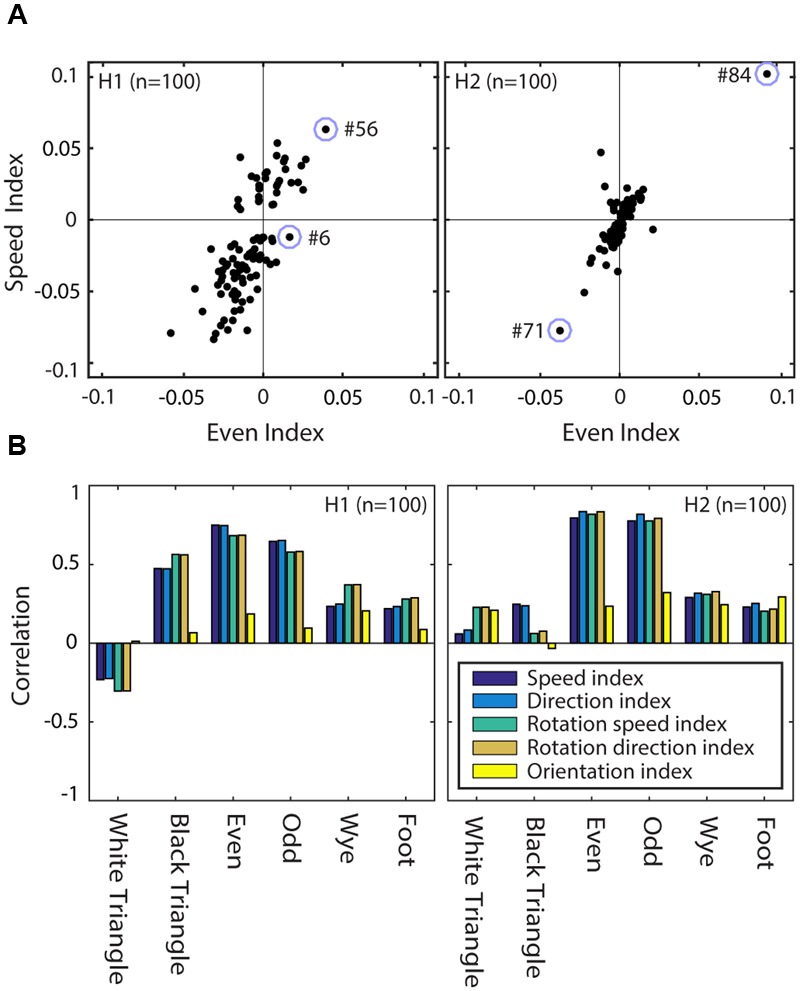
**Texture and motion tuning are correlated in RFAMpa hidden units. (A)** Speed selectivity (SSIt) versus even texture selectivity (TSIeven) in H1 (left) and H2 (right). The correlation was *r* = 0.75 for H1 and *r* = 0.79 for H2. Open circles indicate the four example hidden units used for **Figure 10**. **(B)** Correlation between texture selectivity (TSI), and selectivity for speed of translation (SSIt), direction of translation (DSIt), speed of rotation (SSIr), direction of rotation (DSIr), and orientation (OSI). Correlations are calculated separately for each of the MSCT classes (horizontal axes) and separately for the units in H1 (left panel) and H2 (right panel). This figure shows that the correlation between texture selectivity and orientation selectivity is generally weak (yellow bars), while the correlation between texture selectivity and selectivity for specific speeds and directions of motion is strong, in particular for the visually salient four-point textures (*even* and *odd*).

**Figure 11A** shows the relationship between the speed selectivity (SSIt) and *even* texture selectivity (TSI*even*) for units of H1 (left panel) and H2 (right panel). Note that most units with a positive (negative) TSI*even* (i.e., they responded more (less) to *even* textures than to *random* textures) had a positive (negative) SSIt (i.e., they responded more (less) to moving stimuli than to stationary stimuli). This is consistent with the examples of **Figure 10**; hidden unit #6 had a weak speed tuned response (small SSIt and SSIr) and small *even* textures response (near zero TSI*even*) while hidden unit #56 in H1 and #84 in H2 had strong positive SSIt and SSIr, as well as strong even texture responses (TSI*even* > 0).

We captured the association between texture selectivity and selectivity for more traditional stimulus features by calculating the (Pearson) correlation between the selectivity indices. For instance, the data in **Figure 11A** show a correlation between TSI*even* and SSIt of *r* = 0.75 for H1 and *r* = 0.79 for H2. The analogous correlation measures between all pairwise combinations of the texture selectivity indices (i.e., TSI for each of the seven MSCT) and the five orientations and motion tuning selectivity indices (OSI, SSIr, SSIt, DSIr, DSIt) are shown in **Figure 11B**. This figure shows two important results. First, the traditional measure of form selectivity (orientation selectivity) was only weakly correlated with multipoint texture tuning (yellow bars). Second, all measures of motion tuning (speed, as well as direction selectivity for translation and rotation) were strongly correlated with texture selectivity for the visually salient four-point textures (*even* and *odd*). This association between motion and complex form was much weaker for the visually salient three-point textures (*white triangle* and *black triangle*), and the non-salient four-point textures (*wye* & *foot*), especially in V2.

#### Robustness

The training algorithm of the artificial neural network includes a random initialization of network connectivity, and the backpropagation algorithm is not guaranteed to find a globally optimal solution. Because of this, one might be concerned that the variety of response dynamics across the network (**Figure 7**) and the emergent property of motion tuning (**Figure 10**) could be artifacts of the training algorithm. To address this, we repeated the full training procedure, with randomly chosen weight initializations 10 times and performed the same analyses as above for each of those networks. The same conclusions could be drawn from each of those model networks, showing that our findings are robust. For instance, repeating the analysis leading to **Figure 9** showed that the first two principal components of each model network were very similar to the components shown in **Figure 9** (average correlation *r* = 0.84), and the convex hull of each of the 10 trained networks overlapped on average 80% with that of the other networks. The same was true for motion tuning which was correlated with selectivity for the 4th order multipoint correlations in all 10 networks (*r* between 0.56 and 0.75 for H1 and 0.8 and 0.87 for H2). Together with the high level of performance on the test set, this shows that the diversity in temporal dynamics and the presence of significant motion tuning are not artifacts of the random network initialization or suboptimal solutions found by backpropagation, but a robust and salient aspect of how this recurrent network model generates selectivity for multipoint correlations.

We also performed a control analysis in which we swapped the V2pa target response to the black triangle and even MSCT classes and then performed the same network training and analysis procedure. In other words, we created a counterfactual model of V2pa that responded more strongly to three-point than to even four-point MSCT. In this network the correlations between motion and form selectivity were all weak (all *r* < 0.25). Specifically, this included the correlation between tuning for the speed and direction of translational motion and the fourth-order even class (H1: *r* < 0.17; H2: *r* < 0.12), and the third-order black triangle class (H1: *r* < 0.02; H2: *r* < 0.19). This shows that the correlation between form and motion tuning does not emerge solely from using a recurrent neural network, but that it requires a recurrent network trained to reproduce the specific dynamic responses observed in V2pa.

As discussed above, focusing on the population average response in the RFAMpa network had several advantages, but we found analogous properties of the hidden units in the RFAM that reproduced the responses of all 123 recorded V2 cells. For completeness, we list them briefly here. First, both hidden layers had units with diverse MSCT selectivity. Second, the hidden units had complex time courses that could largely be explained by the first two PCs shown in **Figure 9** (*r* = 0.87). Third, many hidden units of both hidden layers were tuned to dynamic stimuli and their motion tuning strength was highly correlated with their selectivity strength for the visually salient four-point textures (combined H1 *r* = 0.67, H2 *r* = 0.97) and not the visually salient three-point textures (combined H1 *r* = 0.14, H2 *r* = 0) nor the non-visually salient four-point textures (combined H1 *r* = 0.2, H2 *r* = 0.3). This demonstrates another form of robustness of our results; the self-organized tuning properties of the hidden units occur equally in a network trained to reproduce each of the V2 neurons (RFAM), or a network trained to reproduce only the average supragranular V2 MSCT response (RFAMpa).

## Discussion

We developed a novel network model with two recurrently connected hidden layers to explain the response properties of V2 neurons to complex spatial patterns. This model captured texture tuning, generalized to new stimulus examples from the texture classes, and reproduced not only the mean FR, but also the temporal dynamics of the neural responses.

Analyzing the hidden units of the RFAM revealed that the layers of the model self-organized in a hierarchical fashion similar to V1 and V2. Specifically, texture tuning was more pronounced in the second hidden layer than the first hidden layer, analogous to the tuning difference between V2 and V1. Moreover, the dynamic responses of the hidden units in H1 and H2 were highly diverse but quantitatively most similar to those observed in V1 and V2, respectively.

Most importantly, our analysis led to the experimental prediction that signals representing complex form and motion originate from the same early visual neurons.

### LN versus RFAM

Our first attempt to model multipoint selectivity made use of a standard and rather general approach that seeks to capture neural responses as a sum of LN filters (Pillow and Simoncelli, 2006). Given that this method has been used successfully across a range of visual processing stages (Chichilnisky, 2001; Rust et al., 2005; Richert et al., 2013), we reasonably expected it to provide insight into the processing of multipoint correlations. However, while an acceptable fit to training sets could be obtained, predictions of the fitted models failed to generalize – i.e., they did not properly predict responses to stimulus examples outside of the training set (**Figure 4**). This failure to generalize indicates that the training set was overfit.

There are a number of incremental changes one could make to the LN approach in an effort to improve its generalization performance such as adding higher-order filters, estimating non-separable high-dimensional non-linearities, estimating space-time instead of space-only filters, or adding a second stage in which filters are combined non-linearly (Rust et al., 2005). However, given that the simple LN model already captured the training data well, it seems likely that these additions – which add significant complexity but retain the core structure and parameters of the LN model – would merely increase overfitting.

We believe this to be an important general point. A stack of LN channels always captures some fraction of the variance in an experimental data set, and – given a sufficient number of channels – one can approximate any transformation. Sometimes, the filters provide an intuitive way to understand the input–output mapping (e.g., oriented filters for neurons with orientation selectivity (Ts’o et al., 1986) or space-time oriented filters for neurons with motion tuning (Rust et al., 2005). However, there is no guarantee that this is the case and our analysis warns against a mechanistic interpretation of such filters. Filters are informative only if they generalize to new examples from the same class (e.g., other oriented patterns, other moving patterns) or if they generate novel predictions that can be confirmed experimentally (Rust et al., 2005). Without such confirmation of generalization, the model does not provide insight into the underlying computational mechanisms.

The RFAM, on the other hand, generalized well out-of-sample. The RFAM approach differs from the LN approach in many ways, making it difficult to isolate the reason for their contrasting performances. Nevertheless, it is instructive to consider which factors contributed to the better generalization in the RFAM approach.

First, we trained the RFAM network on the full time course while we used only the mean FR to determine the LN model parameters. While one could extend the LN model with spatiotemporal filters (as in previous work Rust et al., 2005; Hartmann et al., 2011; Richert et al., 2013), estimating space-time filters would lead to even worse overfitting – as it would add free parameters more rapidly than it would add constraints. Here, because the stimuli were all unmodulated in time, and the time course of each neuron’s response is similar across MSCT classes (Yu et al., 2015), even the restriction to space-time separable filters would suffer from this problem. In a recurrent network, however, the intrinsic dynamics predict a time course and adding time points to the to-be-explained data increases the constraints on the model without increasing the number of free parameters. These additional constraints reduce the tendency to overfit the data.

Second, we trained a single RFAM network to generate the output of all V2 neurons simultaneously, whereas the LN approach determines a separate, independent filter for each V2 neuron. Forcing a set of hidden units to generate a representation that results in well-matched output of all V2 neurons likely reduced overfitting the noise in the response of any single V2 neuron. Incorporating this approach in the LN model would lead to a feedforward network with a single hidden layer and an output layer representing, for instance, all V2 neurons. While it is possible that such a model would also generalize to new patterns however, capturing the time-course of the response would still require adding time delays, which, as explained above, increases the number of free parameters in an LN model and thereby the risk of overfitting.

### Models and Inference

The comparison of the LN and RFAM models leads us to a more general comment about the limitations of inferences one can draw from a successful model. Our recurrent model captured the experimental data and generalized to new samples, while the feedforward model did not. Can one infer from this that recurrent connections are necessary to capture complex form processing? No, the model only shows that they are sufficient, and we will argue that sufficiency is all a model can ever show.

The universal approximation theorem (UAT) proves that feedforward networks with a single hidden layer can approximate any input-output mapping (Hornik et al., 1989). This implies that a feedforward network exists that can perform just as well as the RFAM. Similarly, the RFAM performance cannot be used to argue that its two recurrent layers are necessary because a single layer recurrent network is also a universal approximator (Funahashi and Nakamura, 1993). In other words, goodness of fit, or lack of such fit, cannot be taken as evidence to support the necessity for recurrent connections, nor the need for two layers. In fact, the choice between network architectures can never be based on the performance of the network alone. Instead, such choices must be based on other, more subjective or domain-specific aspects of the modeling approach.

For instance, there are practical matters such as the ease with which a solution can be found in a specific architecture (the UAT guarantees that a solution exists, but there are no algorithms that are guaranteed to find this solution). Here, simpler feedforward models have a clear advantage as there are reliable methods to find optimal solutions (Pillow and Simoncelli, 2006). Second, a feedforward network maps input sequences to neural responses by using spatiotemporal weights that allow each neuron to look back in time to previous inputs. This convenient short cut punts on the mechanistic question how a network integrates information over time. In our view, this question is of great interest, and this forces us to look beyond feedforward networks. Third, *a priori* knowledge can motivate one model over others. In the current context, the ubiquity of recurrent connections in the brain suggests that recurrent network models are ultimately more viable descriptions of brain function than feedforward models.

These considerations motivated us to develop a model based on recurrent connections, and our results show that it performed better than existing feedforward models. The true value of our model, however, is not that it captured the data better (many models could do that), but that it leads to a novel mechanistic hypothesis of the computations underlying higher-order form processing (below), and testable predictions about the relationship between form and motion processing in early visual cortex (**Figure 11**). In our view, models are best thought of as hypotheses; their value resides in the novelty of the insight they generate and the testability of their predictions.

### Form and Motion

Why would motion and form analysis go hand-in-hand? Motion detectors can be characterized as logical-and operations: a moving object was here at this time *and* there some time later. Four-point correlations can similarly be detected as the logical-and of two orthogonal orientations. Consistent with this, many V2 neurons appear to have sensitivity to orthogonal orientations (Anzai et al., 2007). As our analysis of the LN model shows, however, feedforward solutions in which the logical-and is computed using high thresholds do not generalize well across the textures in a class.

We therefore propose that recurrent connections provide a robust way to compute a logical-and (Salinas and Abbott, 1996) while also providing a rudimentary memory that allows the comparison or integration of neural output at different times (Joukes et al., 2014; Quiroga et al., 2016). The duration of this memory, or the effective integration time of (parts of) the network, can be adjusted by the strength of the recurrent connections (Quiroga et al., 2016; Pachitariu and Sahani, 2017). This flexibility allows the network to detect first- and second-order statistics in one part of the texture and compare this with first- and second-order statistics in one or more other parts of the image after a short delay. For images that are presented abruptly and then remain static during the delay, this comparison will yield selective responses to specific third- and fourth-order spatial statistics. For images that translate in time, this will yield sensitivity to motion patterns, including those driven by high-order statistics (Chubb and Sperling, 1988; Clark et al., 2011). This sketch of the potentially underlying computations directly leads to the prediction that motion and texture tuning are intricately entwined in early visual cortex.

At face value this prediction appears to be at odds with the view that form and motion processing proceed along largely independent pathways in the brain (Livingstone and Hubel, 1984; Hubel and Livingstone, 1987). However, such claims are typically based on the lack of correlation between tuning for orientation and tuning for motion. This correlation is also low in the hidden units of the RFAM network (H1 *r* = 0.31, H2 *r* = 0.37), but orientation tuning is only one aspect of form selectivity: our analysis predicts specifically that selectivity for four-point correlations should correlate strongly with motion tuning, especially in V2 (**Figure 11**).

In addition, anatomical evidence shows a significant degree of convergence of form and motion processing in V1 (Fitzpatrick et al., 1994; Callaway and Wiser, 1996; Sawatari and Callaway, 2000) as well as V2 (Sincich and Horton, 2002). These connections may provide the substrate for our predicted interaction. Moreover, the perceptual and neural interactions between complex shapes and motion in early and mid-level visual areas (Kourtzi and Kanwisher, 2000; Krekelberg et al., 2003, 2005; Kourtzi et al., 2008) generally support the link between motion and form processing. Our current hypothesis, however, is more specific and awaits a direct experimental test.

The relative paucity of motion selective responses in V1 and V2 (e.g., Orban et al., 1986; Lu et al., 2010) may reduce enthusiasm to perform such tests. Indeed, quantifying selectivity in the model is much easier than in an experiment because the model has no noise, and because we can record from model units for virtually unlimited time. We are currently developing closed-loop methods that use the model of a specific neuron to predict which specific subset of moving patterns would be most diagnostic for that neuron. This will greatly reduce the experimental time needed to test our hypothesis. In addition, it may be possible to exploit the fact that motion selectivity is not uniformly distributed across cortex (e.g., more motion selectivity in the thick stripes of V2; Hubel and Livingstone, 1987). Based on this one would expect that the spatial organization of four-point selectivity could also be non-uniform. Optical imaging experiments analogous to the work of (Lu et al., 2010) could test this prediction.

## Conclusion

A network with two recurrently connected hidden layers captured the selectivity of V1 and V2 neurons for multipoint correlations and generalized to new examples from the texture classes. Analysis of this network shows that perceptually salient four-point correlations can be detected by a network with diverse selectivity for all MSCT texture classes and with complex time courses that closely match the properties of V1 and V2 neurons. In this network, many units were motion tuned and the extent of motion tuning was correlated with tuning for the perceptually salient spatial multipoint correlations. This leads to the prediction of a specific overlap between tuning for complex form and motion in early visual processing.

More broadly, our work shows that recurrent connectivity – a defining characteristic of all cortical networks – can solve computational problems in unexpected ways. We trained an artificial recurrent neural network to capture the full time course of the neural response to a sensory input and, in doing so, uncovered a new neural solution to a complex computational problem. Because the artificial network can be probed in depth and at length, it lends itself well to generate novel and experimentally testable predictions. We believe that this approach is a useful method to uncover novel computational principles well beyond early visual cortex.

## Author Contributions

Conceptualization: JJ and BK; Methodology: JJ; Software: JJ; Formal Analysis: JJ; Investigation: JJ and YY; Data Curation: JV and BK; Writing – Original Draft: JJ and BK; Writing – Review and Editing: BK and JV; Visualization: JJ; Supervision: BK and JV; Project Administration: BK and JV; Funding Acquisition: BK and JV.

## Conflict of Interest Statement

The authors declare that the research was conducted in the absence of any commercial or financial relationships that could be construed as a potential conflict of interest.
